# Diagnostic Value of Endoscopic Ultrasound after Neoadjuvant Chemotherapy for Gastric Cancer Restaging: A Meta-Analysis of Diagnostic Test

**DOI:** 10.3390/diagnostics12010100

**Published:** 2022-01-03

**Authors:** Victor Mihai Sacerdotianu, Bogdan Silviu Ungureanu, Sevastita Iordache, Adina Turcu-Stiolica, Antonio Facciorusso, Stefano Francesco Crinò, Adrian Saftoiu

**Affiliations:** 1 Gastroenterology Department, University of Medicine and Pharmacy of Craiova, 200349 Craiova, Romania; sacerdotianumihai@gmail.com (V.M.S.); bogdan.ungureanu@umfcv.ro (B.S.U.); adriansaftoiu@gmail.com (A.S.); 2 Pharmacoeconomics Department, University of Medicine and Pharmacy of Craiova, 200349 Craiova, Romania; 3 Pancreas Center, Gastroenterology Unit, Department of Medicine, University of Verona, 37134 Verona, Italy; antonio.facciorusso@virgilio.it (A.F.); stefanofrancesco.crino@aovr.veneto.it (S.F.C.); 4 Department of Medical and Surgical Sciences, Section of Gastroenterology, Ospedali Riuniti di Foggia, 71122 Foggia, Italy

**Keywords:** endoscopic ultrasound, gastric cancer, restaging

## Abstract

This study aimed to evaluate the diagnostic value of endoscopic ultrasound (EUS) after neoadjuvant therapy (NT) for gastric cancer restaging by meta-analysis. We conducted a systematic search of studies published on PubMed and Web of Science up to 30th August 2021. Assessing the risk of bias in the included studies was done with the QUADAS-2 tool. We used R and Review Manager 5.4.1 for calculations and statistical analysis. To evaluate the diagnostic value of EUS after NT for gastric cancer restaging, we performed a meta-analysis on six studies, with a total of 283 patients, including true-positive, true-negative, false-positive, and false-negative results for T1-T4, N0. EUS as a diagnostic test for GC patients after chemotherapy has a relatively low DOR for the T2 (3.96) and T4 stages (4.79) and a relatively high partial AUC for the T2 (0.85) and T4 (0.71) stages. Our results reveal that the pooled sensitivity for T stages after chemotherapy is rather low (29–56%), except for the T3 stage (71%). A potential limitation of our study was the small number of included studies, but no significant heterogeneity was found between them. Our meta-analysis concludes that EUS is not recommended or is still under debate for GC restaging after NT.

## 1. Introduction

Gastric cancer (GC) is still one of the most common malignancies and remains a significant health problem. Despite all diagnostics and therapeutic progresses, GC was responsible for over one million new cases and more than 750,000 deaths worldwide in 2020 as it ranks fifth for incidence and fourth for mortality worldwide [1]. Accurate tumor assessment [2] as well as biomarkers [3] may lead to an appropriate treatment and better outcomes for patients with GC. Endoscopic ultrasound (EUS) is a reliable tool for the preoperative staging of these patients. The last edition available of the American Joint Committee on Cancer and the Union for International Cancer Control (AJCC/UICC) is widely used for disease staging and therefore to guide the most effective treatment [4]. TNM staging accuracy, sensitivity, and specificity for EUS were intensively studied for this type of cancer due to its strong influence on the treatment decision [5,6] and at the same time to the clear association with disease prognosis [7]. Endoscopic resection and surgery are recommended for patients with early GC. Unfortunately, most cases of GC are found with advanced locoregional disease or in a metastatic stage. Patients with locoregional diseases are candidates for surgery, whether that is alone or associated with neoadjuvant therapy (NT), when the tumor stage is cT2 or higher and is surgically resectable. Neoadjuvant chemotherapy leads to cancer downstaging and facilitates surgical resection which improves progression-free and overall survival (OS) [8,9]. An accurate evaluation of tumor response after NT is mandatory for the correct assessment of resectable or unresectable status. National Comprehensive Cancer Network guidelines recommend contrast computed tomography or FDG-PET/CT in the assessment of GC response to preoperative chemoradiation [10].

Despite EUS being an adequate method for initial locoregional staging (uTNM) [11] for GC, data about EUS utility in disease restaging after NT (yuTNM) are scarce. The use of EUS for post-NT restaging was studied in the past for esophageal cancer and GC, and only a moderate accuracy is attributed to this method [12]. A pertinent question is if EUS can reach a good performance for the post-NT evaluation of patients with GC. Few studies that evaluate the accuracy of EUS post-NT are available and, to our knowledge, no systematic review or meta-analysis of this topic exists. This meta-analysis aimed to demonstrate the value of EUS for preoperative classification after NT on patients with GC.

## 2. Materials and Methods

### 2.1. Literature Search Strategy

The process of the literature search and study selection was performed according with the updated guidelines of PRISMA 2020 [13]. A systematic search was executed using the databases PubMed and Web of Science up to 30th August 2021. The inclusion criteria involved the keywords ((endoscopic ultrasound) OR (endoscopic ultrasonography) OR (EUS)) AND ((gastric cancer) OR (gastric adenocarcinoma) OR (stomach cancer)) AND ((Neoadjuvant) OR (NT) OR (Preoperative)) AND ((Restaging) OR (Response) OR (Relapse)) in PubMed and ((TS = (Gastric cancer OR gastric adenocarcinoma OR stomach cancer)) AND TS = (endoscopic ultrasonography OR endoscopic ultrasound OR EUS)) AND TS = (Neoadjuvant OR NT OR Preoperative) AND TS = (Restaging OR Response OR Relapse) in Web of Science. The referenced studies were also screened to identify other eligible studies.

### 2.2. Selection Criteria

The selected inclusion criteria were based on the PICOS principle: (1) Participants: adults with gastric cancer (adenocarcinoma); (2) Interventions: EUS performed before surgery on patients who received NT; (3) Comparisons: the reference standard (confirmation by histopathological analysis of surgical specimens); (4) Outcomes: data for reporting/calculating true-positive (TP), true-negative (TN), false-positive (FP), and false-negative (FN) results; (5) Study design: diagnostic research with index text (EUS). The included studies were prospective or retrospective, cross-sectional studies, or randomized clinical trials. Both sexes for patients with no age limit were included. We accepted the criteria stated by the authors to classify the T and the N staging, which is from the fifth edition to the seventh edition of the TNM classification and planned to explore it as a source of heterogeneity.

Exclusion criteria were as follows: (1) studies that involved animals and/or ex vivo samples; (2) other types of gastric tumors than gastric adenocarcinoma (mesenchymal tumors, lymphoma); (3) studies investigating only GEJ/cardia cancer with no tumors from other sites of the stomach (different behavior); (4) patients without neoadjuvant therapy received before surgery; (5) contrast agent or miniprobes used for EUS restaging; (6) studies of low methodological quality; (7) case series, review articles, meta-analyses, abstracts, or letters; (8) literature with insufficient data; and (9) studies published in a language other than English.

### 2.3. Quality Assessment

The quality of the included studies was evaluated using the QUADAS-2 (Quality Assessment of Diagnostic Accuracy Studies-2) tool [14]. The four domains (patient selection, index test, reference standard, and flow and timing) were used to objectively evaluate the risk of bias and the preoccupations about the applicability of the included studies. Two review authors (BSU and VMS) independently screened the quality of studies, and the differences were arbitrated by a third author (A.T.-S.).

### 2.4. Data Extraction

Two investigators (BSU and VMS) independently extracted the information from all eligible studies: the first author, the year of publication, the research country, TP, TN, FP, and FN. The disagreements between the two investigators were settled by discussion till an agreement was reached with the third investigator (A.T.-S.). Some of the articles reported directly diagnostic accuracy measures (TP, TN, FP, and FN), and others needed to be calculated from sensitivity, specificity, positive predictive value, and accuracy [15]. For two studies, an email was sent to the correspondence author to find the not-reported measures [16,17], but with no received answers.

### 2.5. Statistical Analysis

We performed a statistical analysis with RevMan 5.4.1 software (The Cochrane Collaboration, 2020) and mada R-package (R foundation, Vienna, Austria). Pooled sensitivity and specificity were plotted using a summary receiver operating characteristic (SROC) curve to explore the performance of EUS for T1, T2, T3, T4, T1 + T2, and N0 after receiving neoadjuvant therapy, using a bivariate random-effects model and a Bayesian approach. Area under ROC curve (AUC) and the partial AUC (using only the region where false-positive rates of studies were actually observed and then normalized to the whole space) were calculated to evaluate the overall accuracy (a value higher than 0.75 represents high diagnostic efficacy). A favorite test has an AUC close to 1, while a weak test has an AUC close to 0.5. The pooled diagnostic odds ratio (DOR), correlation between sensitivities and false positive rates, and their corresponding 95% confidence intervals (CIs) were also obtained to estimate a prediction region where future pairs (sensitivity and specificity) are expected to be found [18]. High heterogeneity was demonstrated for higher value of Higgins *I*^2^ (an *I*^2^ greater than 50% was suggestive of substantial heterogeneity). Heterogeneity of sensitivities and specificities were evaluated using χ^2^ test, the null hypothesis being that all are equal for all the included studies. The bivariate random-effects model was performed if there was heterogeneity between studies; otherwise, the fixed-effects model was used. A *p*-value less than 0.05 was considered statistically significant.

## 3. Results

### 3.1. Electronic Search Results and Study Characteristics

**Figure 1:**
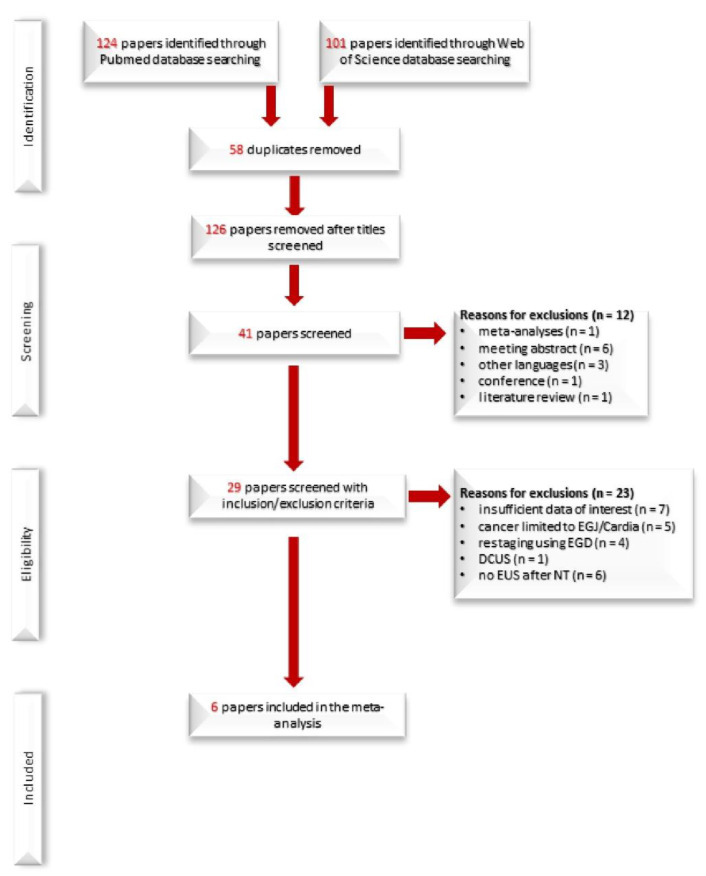
Study flow PRISMA diagram.

According to the search protocol, we finally included six studies involving 285 patients. The characteristics of the included studies are included in Table 1. The flow diagram of the literature search and study selection according to PRISMA statement is detailed in Figure 1.

### 3.2. Quality Assessment of the Included Studies

**Figure 2:**
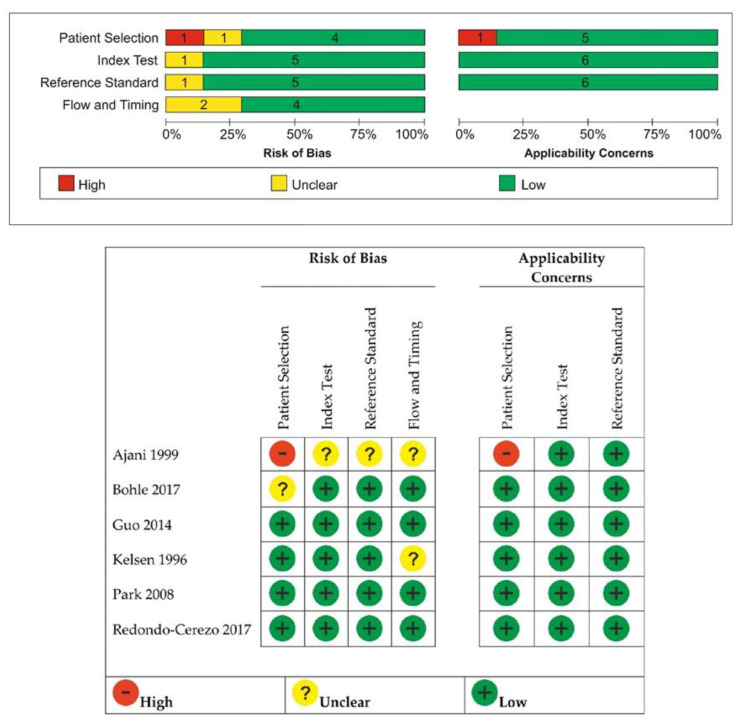
The review authors’ judgment about each domain of bias and applicability concerns across the included studies.

We found a high risk of bias in the domain of “Patient Selection” after the quality assessment using the QUADAS-2 tool for only one study [19] that did not avoid inappropriate exclusions. Only two studies [19,22] from all six introduced bias with patient flow and timing, having an unclear appropriate interval between the index test and the reference standard, as in Figure 2. All assessed domains exhibited low concerns regarding their applicability.

### 3.3. Data Synthesis

#### 3.3.1. T1 Restage

**Figure 3:**
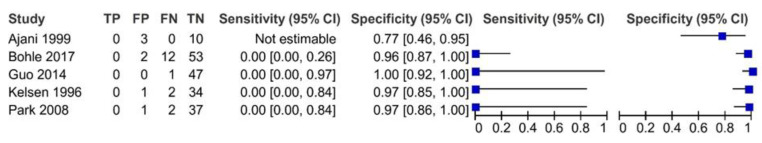
Forest plot for T1 restaging.

Five studies reporting 246 patients were included for this test. The pooled diagnostic test accuracy was not possible to be merged because the sensitivities were 0 or not estimable, as in Figure 3.

#### 3.3.2. T2 Restage

**Figure 4:**
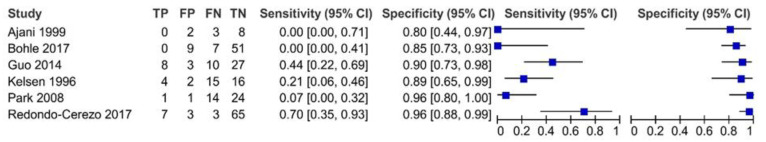
Forest plot for T2 restaging.

**Figure 5:**
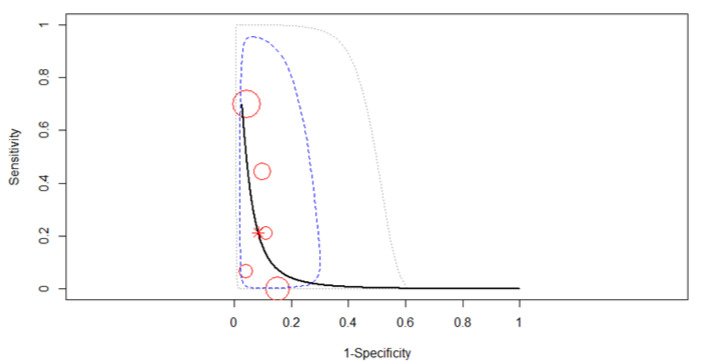
SROC curve for T2 restaging. Dotted blue curve: 95% confidence region; dotted closed curve: 95% prediction region for T2 staging.

The forest plot below in Figure 4 shows the studies in alphabetical order. All six studies were merged to derive pooled diagnostic test accuracy using a fixed-effects model. The SROC curve is shown by the black solid curve through the estimated mean (sensitivity, false positive rate) (0.29, 0.11). The pooled sensitivity was 0.29 (95% CI, 0.11–0.57). The large heterogeneity of sensitivities as compared to the small heterogeneity in specificities is clearly visible in Figure 5. Different sensitivities were found between the studies (χ^2^ = 15.4, *p* = 0.0039). The pooled specificity was 0.89 (95% CI, 0.83–0.94). The same specificities were found between the studies (χ^2^ = 4.77, *p* = 0.31). A high diagnostic efficacy was found with the AUC of 0.85. The partial AUC was 0.25, which is much smaller. The difference alerts us to the fact that the region in which the observed data lies is rather narrow, so we have limited direct knowledge about the data and the shape of the overall ROC curve. No significant heterogeneity between studies was found (Tau^2^ = 1.66, *I*^2^ = 6.34%, *p* = 0.37). DOR (95% CI) was 3.96 (0.95–16.62).

#### 3.3.3. T3 Restaging

**Figure 6:**
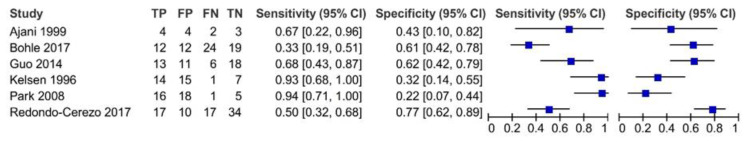
Forest plot for T3 restaging.

**Figure 7:**
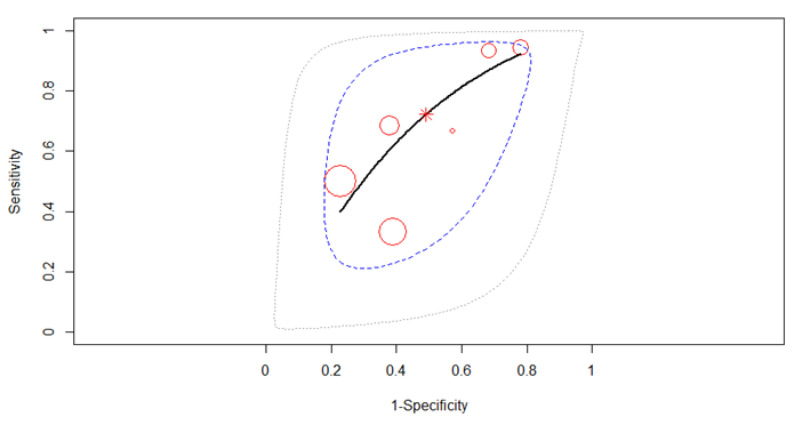
The SROC curve for T3 gastric cancer restaging.

All six studies were merged to derive pooled diagnostic test accuracy using a random-effects model. The most striking feature of the forest plot below (Figure 6) is the greater uncertainty (indicated by the confidence interval width). The SROC curve in Figure 7 is shown by the black solid curve through the estimated mean (sensitivity, false positive rate) (0.71, 0.51). The pooled sensitivity was 0.71 (95% CI, 0.45–0.89). Different sensitivities were found between the studies (χ^2^ =27.91, *p* < 0.0001). The pooled specificity was 0.49 (95% CI, 0.31–0.68). Different specificities were found between the studies (χ^2^ =25.39, *p* = 0.0001). A moderate diagnostic efficacy was found with the AUC of 0.62. The partial AUC was 0.69. Significant heterogeneity between studies was found (Tau^2^ = 0.18, *I*^2^ = 76.4%, *p* = 0.03). DOR (95% CI) was 2.28 (1.08–3.46).

#### 3.3.4. T4 Restaging

**Figure 8:**
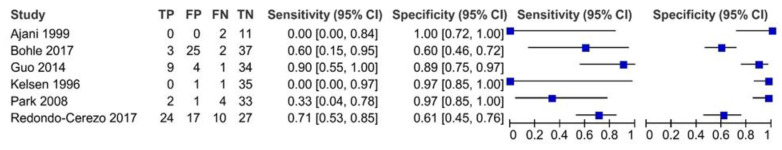
Forest plot for T4 restaging.

**Figure 9:**
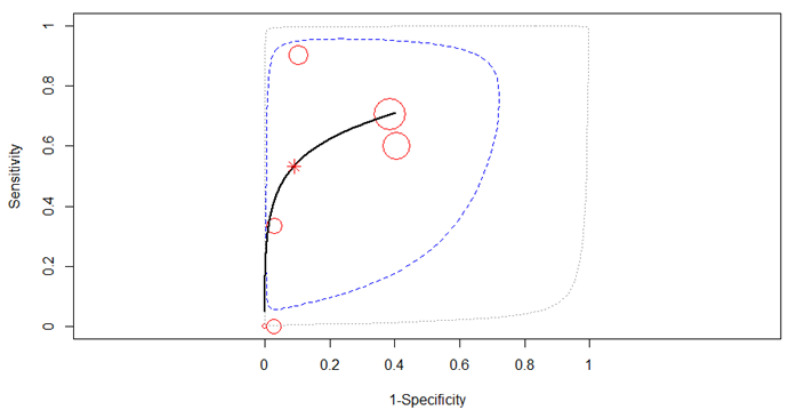
SROC curve with 95% confidence and prediction regions for T4 restaging.

All six studies were merged to derive pooled diagnostic test accuracy using a random-effects model, as in Figure 8. The SROC curve in Figure 9 is shown by the black solid curve through the estimated mean (sensitivity, false positive rate) (0.56, 0.13). The pooled sensitivity was 0.56 (95% CI, 0.37–0.72). The same sensitivities were found between the studies (χ^2^ = 9.69, *p* = 0.08). The pooled specificity was 0.87 (95% CI, 0.67–0.95). Different specificities were found between the studies (χ^2^ = 37.97, *p* < 0.0001). A moderate diagnostic efficacy was found with the AUC of 0.71. The partial AUC was 0.58. No significant heterogeneity between studies was found (Tau^2^ = 0.43, *I*^2^ = 6.34%, *p* = 0.37). DOR (95% CI) was 4.79 (0.43–6.33).

#### 3.3.5. T1+T2 Restaging

**Figure 10:**
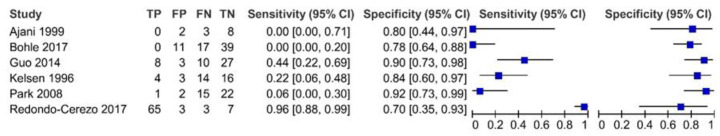
Forest plot for T1 + T2 restaging.

**Figure 11:**
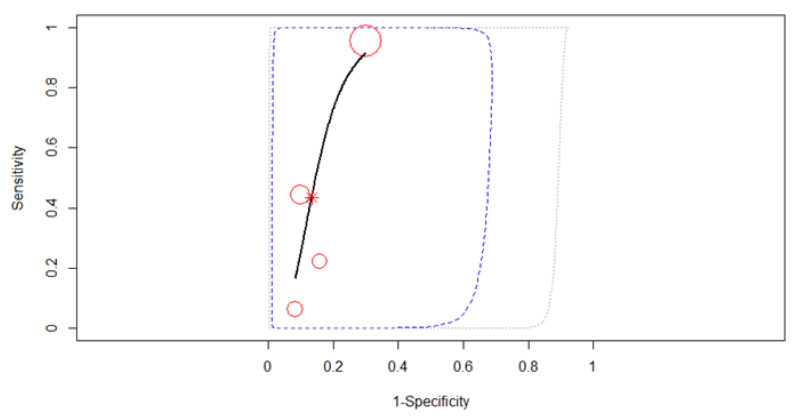
SROC curve with 95% confidence and prediction regions for T1+T2 restaging.

All six studies were merged to derive pooled diagnostic test accuracy using a fixed-effects model, as in Figure 10. The SROC curve in Figure 11 estimated mean (sensitivity, false positive rate) (0.45, 0.14). The pooled sensitivity was 0.45 (95% CI, 0.07–0.89). Different sensitivities were found between the studies (χ^2^ = 70.06, *p* < 0.0001). The pooled specificity was 0.86 (95% CI, 0.72–0.94). The same specificities were found between the studies (χ^2^ = 3.33, *p* = 0.34). A high diagnostic efficacy was found with the AUC of 0.84. The partial AUC was 0.65. No significant heterogeneity between studies was found (Tau^2^ = 2.22, *I*^2^ = 5.84%, *p* = 0.47). DOR (95% CI) was 4.8 (2.02–6.93).

#### 3.3.6. N Restage (N0 vs. N1+)

**Figure 12:**
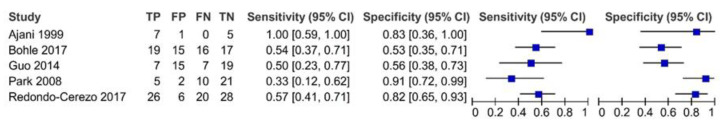
Forest plot for N0 restaging.

**Figure 13:**
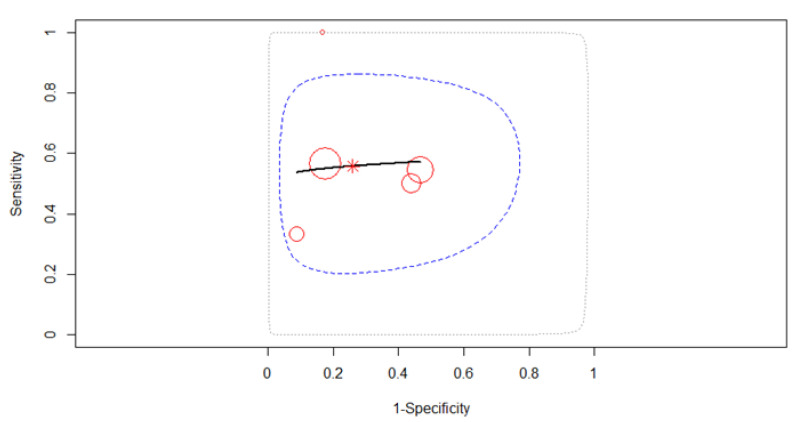
SROC curve describing the diagnostic performance of EUS in N0 restaging.

Five studies reporting data on 246 patients were included in the meta-analysis, as in Figure 12. The SROC curve in Figure 13 estimated mean (sensitivity, false positive rate) (0.53, 0.28). Since no heterogeneity was identified in our meta-analysis (Tau^2^ = 0.562, *I*^2^ = 13.49%), a fixed-effects model was applied for the pooled analysis. The pooled sensitivity was 0.53 (95% CI, 0.44–0.62) with similar values between the sensitivities of the five studies (χ^2^ = 7.77, *p*-value = 0.1). The pooled specificity was 0.72 (95% CI, 0.53–0.85), the specificities of the five studies being significantly different (χ^2^ = 14.41, *p*-value = 0.006). A small AUC was estimated at 0.55, almost the same as the partial AUC (0.52). The value of the Spearman correlation coefficient rho of sensitivities and false positive rates was −0.005 (95% CI, −0.88 to 0.88) in the threshold effect analysis, suggesting the existence of a threshold effect, which might be the main source of heterogeneity in the present meta-analysis.

Pooled sensitivity and specificity, AUC and partial AUC for T and N restaging are summarized in Table 2.

## 4. Discussion

GC requires a proper imaging assessment and is mandatory to establish a patient’s prognosis. Depending on the TNM stage, GC may benefit from various treatment techniques such as endoscopic resection, surgery, and/or systemic therapy. Recent guidelines recommend that for locoregional disease, cT2, or higher stages, surgery alone is less efficient if NT therapy is not associated. Therefore, pre- and postoperative chemotherapy is the treatment of choice for the management of locally advanced GC [8,10].

Several imaging techniques have been suggested for GC initial assessment before NT, such as magnetic resonance imaging (MRI), multidetector computed tomography (MDCT), 18 F-fluorodeoxyglucose positron emission tomography (FDG-PET/CT), and EUS [24]. Nonetheless, MDCT has proven to have a high accuracy in the detection of tumor invasion, either limited to the gastric wall or extended to adjacent organs. Unfortunately, lymph node involvement can be misdiagnosed as inflammatory lymph nodes. Increasingly used in recent years, MRI has a higher capacity for the characterization of the gastric wall stratification, especially using functional techniques such as diffusion-weighted imaging (DWI), intravoxel incoherent motion (IVIM), and dynamic contrast-enhanced (DCE) imaging. On the other hand, EUS remains the recommended method for tumor invasion assessment due to its high accuracy in describing all five layers of the gastric wall and therefore indicating the cT stage for GC. EUS might also aid in describing the N stage, using parameters like the echogenicity, shape, size, and number of lymph nodes and also by puncturing the lymph nodes. Currently, all guidelines [10,25] recommend EUS and MDCT for the first assessment of GC staging; however, there is no consensus about the indication for GC restaging after NT.

This meta-analysis tries to highlight the EUS findings when considering restaging GC after oncologic treatment by assessing the T and N stage. Our results reveal that the pooled sensitivity for the T stage after NAC is rather low (29–56%), except for the T3 stage (71%), whereas the specificity of EUS for the T stage after NAC is high (72–87%), with the exception of the T3 stage (49%). EUS as a diagnostic test for GC patients after NAC has a relatively low DOR for the T2 (3.96) and T4 stages (4.79). EUS also has a relatively high partial AUC for the T2 (0.85) and T4 (0.71) stages. A high diagnostic efficacy was found when comparing T1 + T2 vs. T3 + T4 with an AUC of 0.84, pooled sensitivity of 45%, and pooled specificity of 86%.

The NAC objective on GC is to reduce the tumor size and, as a result, it may cause inflammation and local fibrosis. Thus, the EUS technique, which requires excellent visualization of the gastric layers to determine the T stage, might be hampered since the local architecture could be distorted. The nearby structures as well as the layers could be fibrotic, with residual tumor tissue, which may suggest a different T stage than the real one. The studies included in the meta-analysis confirmed that both downstaging and upstaging [20,21,22,23] might be encountered. A similar process was also described in esophageal and colorectal cancer [26,27]. Downstaging was observed especially from T4 to T3 [20,21,23], but it was also mentioned for T3 to T2 [21] and even T4–T2 [20], while upstaging was rarely mentioned [17,19,22]. Our analysis revealed that the sensitivity for T3 tumors was acceptable at 71%, which might actually be related to the tumor volume. In addition, the partial AUC for T2 and T4 should be taken into account.

When discussing the N stage, the performance of EUS after NAC is unsatisfactory. The pooled sensitivity and specificity are 53% and 72%, respectively. The AUC is 0.55 and the DOR of the N stage is only 2.97 (95% CI, 1.19–7.44). While some studies proved that a better accuracy might be encountered when comparing EUS to other imaging techniques, such as MDCT or PET-CT, for restaging purposes, covering the N stage does not offer new significant information. Obviously, it is difficult to count all lymph nodes by EUS, mainly because it is difficult to cover all areas but also because some lymph nodes might be misinterpreted as non-malignant. When covering EUS restaging of the lymph nodes by EUS, we might expect a decrease in size due to the effect of NAC. While other studies which used PET-CT suggested that a >1 cm lymph node after NAC is inappropriate [28], Guo et al. proposed a smaller size of >0.7 cm, but also obtained similar results. He concluded that even though the tumor size may be reduced, which might suggest a higher sensitivity of EUS, an upstage process would occur, thus, the recommendation might be to perform an EUS-FNA [21]. However, a systematic sampling for cytopathology for all lymph nodes might be inappropriate.

Unfortunately, there are not many studies with EUS post-NAC used for GC restaging probably because of low interest due to large variations between results, which often prove that the technique is not feasible in the restaging of this disease after NAC. More data can be found from studies that include patients with esophageal and rectal cancer [27,29,30,31]. In a study that assessed the role of EUS for patients with esophageal cancer, Mesenas et al. showed high post-NAC T staging accuracy with EUS as compared with CT (66.7% vs. 57.7%) but without statistical significance (*p* = 0.151). Furthermore, N stage accuracy post-NAC with EUS had slightly higher values than with CT (60% vs. 53%) but also without a statistical difference (*p* = 0.256) [27].

While EUS might not be as promising as hoped as a diagnostic test for restaging GC, a patient’s prognosis might be influenced by performing another procedure after oncologic therapy. Hoibian et al. suggested that a thorough EUS liver examination identified the presence of metastases better than the CT scan, thus resulting in a better selection of patients for surgery [16]. On the other hand, Bohle et al. suggested that a wall tumor thickness of <15 mm after NAC, might be considered an independent wall layer recurrence-free factor for patient prognosis [20]. In addition, other studies discussed the EUS tumor size as a predictive factor for overall survival, with chemotherapy performing a tumor shrinkage [32]. However, no guideline recommends these criteria as a possible prognosis factor in GC, mainly due to the anatomic characteristics of the stomach.

Most of the studies included confirmed that the index test was performed by experienced endosonographers, the time between index and reference test was adequate, and both test results were double-blind interpreted, which considerably reduces the risk of bias. The limitations of this meta-analysis were the low number of patients and that only six studies were included, but with no significant heterogeneity between them. From a clinical point of view, patients benefitted from different types of NAC, according to guidelines and technology available at that time. Secondly, the histologic type was not mentioned in all studies, which may contribute to the heterogeneity. A discrepancy between the AJCC editions is clear, with the included studies being related to some differences of the staging systems which were available at the time of the performance, from the fifth to seventh AJCC/UICC TNM. Some studies assessed the discriminative power of survival difference between each TNM stage of gastric cancer and demonstrated a significant difference in 5-year survival between T2 and T3 gastric cancer classified according to the AJCC seventh edition [33,34]. However, our study did not analyze the clinical benefits in accurate prediction of survival but in the diagnosis of gastric cancer.

## 5. Conclusions

Our meta-analysis concludes that EUS is not recommended or is still under debate for GC restage after NT. EUS for EUS GC restaging after NAC has a low sensitivity, especially in early stages. T3 seems to have a higher sensitivity but with a lower specificity, but these results might be influenced by tumor size. Both upstaging and downstaging may be encountered. When considering the N stage, EUS should not be used for restaging lymph nodes because of the low performance and should only be used as a tool at the initial diagnosis.

## Figures and Tables

**Table 1:** The main characteristics of eligible studies in the meta-analysis.

Study	Country	No of Patients (Included/Total)	Age Mean, Range	Gender Male/Female	Location	AICC/UICC TNM Edition
Ajani 1999 [19]	United States of America (Texas)	13/30	56, 33–75	19/11	Proximal 21 Distal 9	5th ed.
Bohle 2017 [20]	Germany	67	61, 29–80	48/19	Gastric 18 Esophago-gastric junction 44 Distal esophagus 5	7th ed.
Guo 2014 [21]	China	48	62, 34–80	33/15	Cardia 9 Distal+proximal 39	NA
Kelsen 1996 [22]	United States of America (New York)	37/60	57, 26–75	40/20	Proximal 31 Distal 28 Linistis Plastica 1	NA
Park 2008 [23]	Republic of Korea	40/44	58, 36–70	30/10	Proximal 6 Distal 27 Whole stomach 7	6th ed.
Redondo-Cerezo 2017 [15]	Spain	80/256	67.6 (SD = 12.1)	178/78	Fundus 26 Body 102 Antrum 100 Lesser curvature/incisura angularis 30	7th ed.

NA, not available; SD, standard deviation.

**Table 2:** Pooled sensitivity, specificity, DOR and AUC for T2-T4, T1+T2 and N stage.

Stage	Pooled Sensitivity (95%, CI)	Pooled Specificity (95%, CI)	Pooled DOR (95%, CI)	AUC Partial AUC (Restricted to Observed FPRs and Normalized)
T2	29% (11–57%)	89% (83–94%)	3.96 (0.95–16.62)	0.85 (0.25)
T3	71% (45–89%)	49% (31–68%)	2.28 (1.08–3.46)	0.62 (0.69)
T4	56% (37–72%)	87% (67–95%)	4.79 (0.43–6.33)	0.71 (0.58)
T1+T2	45% (7–89%)	86% (72–94%)	4.8 (2.02–6.93)	0.84 (0.65)
N	53% (44–62%)	72% (53–85%)	2.97 (1.19–7.44)	0.55 (0.52)

## Data Availability

The authors declare that the data of this research is available from the correspondence author on request.
